# Composition formulas of binary eutectics

**DOI:** 10.1038/srep17880

**Published:** 2015-12-14

**Authors:** Y. P. Ma, D. D. Dong, C. Dong, L. J. Luo, Q. Wang, J. B. Qiang, Y. M. Wang

**Affiliations:** 1 Key Laboratory of Materials Modification (Ministry of Education), School of Materials Science & Engineering, Dalian University of Technology, Dalian 116024, China; 2 Special Glass Key Laboratory of Hainan Province, School of Materials and Chemical Engineering, Hainan University, Haikou 570228

## Abstract

The present paper addresses the long-standing composition puzzle of eutectic points by introducing a new structural tool for the description of short-range-order structural unit, the cluster-plus-glue-atom model. In this model, any structure is dissociated into a 1^st^-neighbor cluster and a few glue atoms between the clusters, expressed by a cluster formula [cluster]glue_x_. This model is applied here to establish the structural model for eutectic liquids, assuming that a eutectic liquid consist of two subunits issued from the relevant eutectic phases, each being expressed by the cluster formula for ideal metallic glasses, i.e., [cluster](glue atom)_1 or 3_. A structural unit is then composed of two clusters from the relevant eutectic phases plus 2, 4, or 6 glue atoms. Such a dual cluster formulism is well validated in all boron-containing (except those located by the extreme phase diagram ends) and in some commonly-encountered binary eutectics, within accuracies below 1 at.%. The dual cluster formulas vary extensively and are rarely identical even for eutectics of close compositions. They are generally formed with two distinctly different cluster types, with special cluster matching rules such as cuboctahedron plus capped trigonal prism and rhombidodecahedron plus octahedral antiprism.

In a typical eutectic reaction, two solid phases transform to or from a single liquid phase at a specific composition and temperature. In spite of the obvious fundamental as well as engineering interests in such alloys, important especially for their low-melting points, the structures and compositions of eutectic liquids remain open issues. It is widely accepted that a eutectic liquid is characterized by topologic and chemical short-range-order clusters that maintain certain similarities with the eutectic phase structures^1^,^2^,^3^,^4^,^5^,^6^,^7^,^8^,^9^. These alloys in liquid state at temperatures not far from the melting point were considered as cluster solutions, where these clusters are self-associated atomic groups or chemically ordered structural units^1^. According to first-principles molecular dynamics simulations for liquid and undercooled eutectic alloys Au-(Si,Ge) at various temperatures^2^, the local structure presents a well-defined chemical short-range order that enhances dissimilar element interactions in contrast with random solid mixture and may explain the high stability of the liquid phase on the basis of preferential Au-(Si,Ge) bonds. However, the available information on the short-range-order structures in eutectic melts is far from complete and the structures and composition rules of eutectics are largely unknown.

Necessarily a short-range-order structural model is required for any understanding about the structures and compositions of the eutectic points. Actually binary eutectic compositions frequently occur near simple composition ratios^10^ such as 8/1, 5/1, 3/1, 2/1 and 3/2. Based on Frank’s conjecture^11^ that icosahedral cluster might be responsible for liquid undercooling, these ratios were tentatively explained as chained arrangements of icosahedral clusters with the lowest solute atom neighbors^12^. These simple ratios were recently addressed^13^ using the dense cluster-packing model for metallic glasses^14^. The eutectic liquid was treated as being composed of efficiently packed solute-centered atomic clusters where are present four topologically distinct atomic sites. Choosing different coordinate close-packing clusters and changing the occupation style of the interstitial solutes, simple ratios of binary eutectics could be reached. A similar model for metallic glasses was also proposed^15^ where shared quasi-equivalent clusters are packed into an icosahedral-like structure, which explained some binary eutectics and metallic compositions near the solid solution side of binary phase diagrams. However, all these efforts fail in interpreting quantitatively the occurrence of eutectics at various and specific compositions. For this objective, a structural model, enabling a quantitative description of short-range orders, is required to describe precisely the eutectic liquid structure. It is reasonable to anticipate that a structural unit, reflecting the characteristic short-range ordering in the liquid, should be present for a specific eutectic composition.

We have developed a so-called cluster-plus-glue-atom model that suits specifically for short-range-order structure descriptions in quasicrystals, amorphous alloys^16^, and solid solutions^17^. In this model, any structure is dissociated into a 1^st^-neighbor coordination polyhedral cluster and a glue-atom part that are situated outside the cluster part at the 2^nd^ neighbors and beyond. Then the structure can be expressed by a cluster formula [cluster]glue_*x*_, where the cluster is the coordination polyhedron representative of the 1^st^-neighbor short-range order of the structure (notice that the term cluster may mean any agglomeration of atoms in a general sense, but here we confine the cluster concept to cover only the 1^st^-neighbor coordination polyhedra), and the glue atoms between the clusters mark the short-range-order feature on and beyond the 2^nd^ neighbors. For a bulk metallic glass, the number of glue atoms is either 1 or 3^16^,^18^. It was further pointed out^19^,^20^ that the total number of valence electrons per unit cluster formula for an ideal bulk metallic glass is universally about 24, so that the cluster formula for a bulk metallic glass resembles the ‘molecular’ unit of a chemical substance^20^. This formula can also be understood as arising from a certain spatial averaging scheme of a complicated disordered structure into a short-range-order structural unit of a dozen of atoms, covering only the first few neighbors, generally 1^st^ and 2^nd^. It should be emphasized that the clusters are assumed to be isolated from each others in metallic glasses, which is necessary to avoid the center-shell type of nearest-neighbor short-range orders to develop into longer-range ones. Solid-solution alloys have been treated in a similar manner, because they are also characterized by chemical short-range orders, and the formulas well explain the industrial specification composition selection, as exemplified by Cu-Zn and Cu-Ni alloys^17^.

In the following, the cluster-plus-glue-atom structural model and the relevant composition formulas for eutectic liquids will first be proposed, and then boron-containing binary eutectics will be fully covered using this model. It will be shown that, at least for normal eutectics whose compositions do not fall too close to the phase diagram terminals, the model applies perfectly, which arrives at formulated eutectic compositions within accuracies below 1 at.% from experimental ones.

## Cluster-plus-glue-atom model for binary eutectic liquids

A binary eutectic alloy liquid normally decomposes in a coupled growth mode into two eutectic phases with distinct composition differences. It is then reasonable to assume that the eutectic liquid would contain two subunits that evolve towards their respective eutectic phases. Thereof we propose the first assumption for modeling the eutectic liquids:

*1) A eutectic liquid is comprised of two subunits issued from the two eutectic phases.*

As already stated, the compositions at which metallic glass stability reaches the maximum are well expressed by cluster formulas out of eutectic/devitrification phases in accordance with the cluster-plus-glue-atom model^18^. Since metallic glasses can be regarded as frozen liquids, their formulas in fact describe some stable liquids that resist crystallization upon solidification. Also metallic glass formation is generally associated with eutectics. Thereof we introduce the second assumption:

*2) Each subunit is described by a cluster formula of ideal metallic glasses, expressed as [cluster](glue atoms)*_*1 or 3*_

A eutectic liquid is then composed of two subunit liquids, each being formulated either by [cluster](glue atoms)_1_ or by [cluster](glue atoms)_3_, so that the final eutectic composition is expressed by a dual cluster formula:


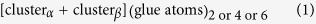


where the two clusters in the brackets belong respectively to the two liquid subunits and are inherited from corresponding eutectic phases α and β.

The eutectic composition interpretation then relies on the identification of the right clusters from the eutectic phases. In explaining a bulk metallic glass composition, the cluster is taken from a known crystalline phase, assuming local structural heritage between the two states. In a given crystal structure, however, there are often multiple nearest-neighbor clusters (centered by any non-equivalent site in unit cell is defined a cluster). Among the multiple clusters, there must be at least one cluster, termed ‘the principal cluster’^21^, that represents the dominating short-range order feature of the structure. This principal cluster should be the most strongly bonded part in the structure, which leads to high cluster isolation and atomic dense packing, as well as high elastic coefficients^22^. The atomic dense packing of a cluster could be measured by the center-to-shell atomic radius ratios, because an ideally densely-packed cluster of certain coordination number (CN) shows a special such ratio^14^. Cluster isolation refers to the cluster size reduction to account for the commonly present cluster overlapping in crystals, and the principal one should show the highest cluster separation.

**Figure 1:**
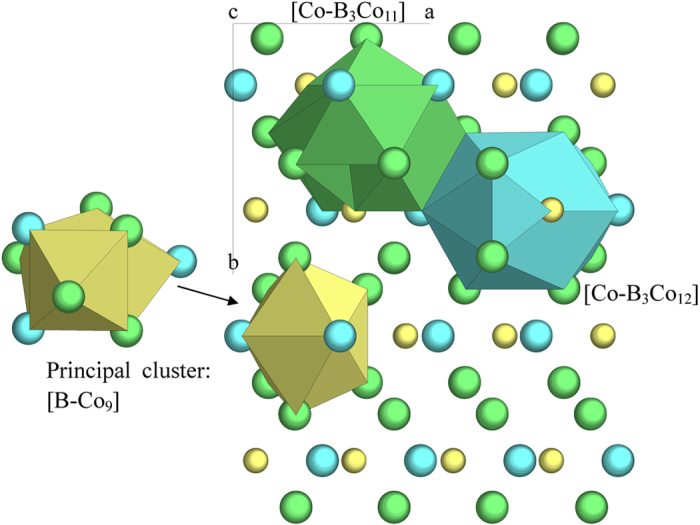
Clusters in BCo_3_ (the CFe_3_ structure). Small spheres represent B and large ones Co. Projection along [001].

For instance, the BCo_3_ phase (CFe_3_ structure type, space group Pnma, the crystal structure data are all from Pearson’s handbook^23^) contains sixteen atoms in its unit cell, which belong to three non-equivalent sites: four B at (0.881, 0.25, 0.431), four Co at (0.044, 0.25, 0.837), and eight Co (0.181, 0.063, 0.337). Centered by these sites are defined three clusters, capped trigonal prism CN9 [B-Co_9_] 
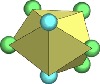
, CN15 [Co-B_3_Co_12_] 
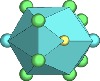
, and CN14 [Co-B_3_Co_11_] 
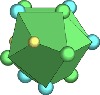
 (Fig. 1). In expressing a cluster, the central atom is placed first and is separated from the nearest-neighbor shell atoms by a hyphen. Their atomic radius ratios of the central atom over that of the shell atoms are respectively 0.70, 1.07, and 1.07, calculated from the B covalent radius of 0.088nm and the Co Goldschmidt radius of 0.125 nm. All being quite close to those of the ideally dense-packed clusters of CN9 (0.71), CN15 (1.12) and CN14 (1.05), the dense packing property alone cannot distinguish effectively the clusters. These clusters are all extensively overlapped with neighboring ones, and the reduced clusters become respectively [B-Co_3_], [Co-B_1_Co_2_], and [Co-B_0.5_Co_0.5_], which are also phase formulas expressed using the three clusters because there is no glue atoms. The cluster reduction rates are respectively 4/9, 3/16, and 1.5/15. Therefore the principal cluster should be CN9 [B-Co_9_] for the highest cluster isolation. This cluster type is frequently encountered in explaining eutectic points.

In the above example, the phase structure is completely occupied by the clusters and the phase formulas expressed using the clusters do not contain the glue atom part. In many cases, such as in B_4_Y, glue atoms are involved. There are four non-equivalent sites in the unit cell of B_4_Y (B_4_Th structure type, space group P4/mbm), four B at (0, 0, 0.2027), four Y at (0.3179, 0.8179, 0), four B at (0.0871, 0.5871, 0.5), and eight B at (0.1757, 0.0389, 0.5). Four clusters are then defined centered by each of the four non-equivalent sites, i.e., [B-B_3_] 
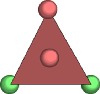
, two square pyramids [B-B_5_] 
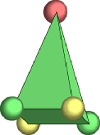
, and [Y-B_18_Y_5_] 
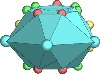
. Among them, only the Y-centered cluster [Y-B_18_Y_5_] gives a satisfactory atomic radius ratio of 1.67, calculated from the central atom B radius of 0.088 nm and the averaged atomic radius B_18_Y_5_ of 0.108 nm (0.18 nm for Y), which compares favorably with the ideal value of 1.61 for the CN23 cluster. The reduced clusters after overlapping are respectively [B-B], [B-B_2_], [B-B_3_], and [Y-B_4_]. In terms of the absolute size, the last one is the largest but in comparison with the complete cluster size, it is not. Such a contradiction actually originates from the fact that glue atoms are involved: the phase formulas expressed by the four clusters are respectively [B-B]B_2_Y, [B-B_2_]BY, [B-B_3_]Y, and [Y-B_4_]. Since the principal cluster should dominate the phase structure, the smallest number of glue atoms are desirable, so that the last one [Y-B_18_Y_5_] is finally selected.

In many cases, the principal clusters can be directly identified from the phase formulas, where the largest phase formula, the smallest cluster size reduction, and the smallest number of glue atoms serve the finger prints for the principal clusters. A phase formula can simply be arrived at by matching the atomic multiplicity of each atom in unit cell. For instance, to reach the phase formula expressed by the [B-Co_9_] cluster, the multiplicity of the central B atom being 4, and those of the two Co non-equivalent sites bring 4 and 8, the reduced cluster becomes [B_1_-Co_1_Co_2_] = [B-Co_3_], which is also the phase formula for the absence of glue atoms here. The use of atomic dense packing is restricted, for atoms are not strictly spherical of constant atomic radii. In the following, the phase formula characteristics are mainly used to determine the principal cluster.

After choosing the principal clusters from two eutectic phases, according to formula (1), one pair of such clusters are matched with two, four, and six glue atoms, giving a dual cluster formula for the eutectic liquid. In the following, B-Co eutectics are explored in detail to exemplify the composition interpretation procedures using the principal clusters from respective eutectic phases.

**Figure 2:**
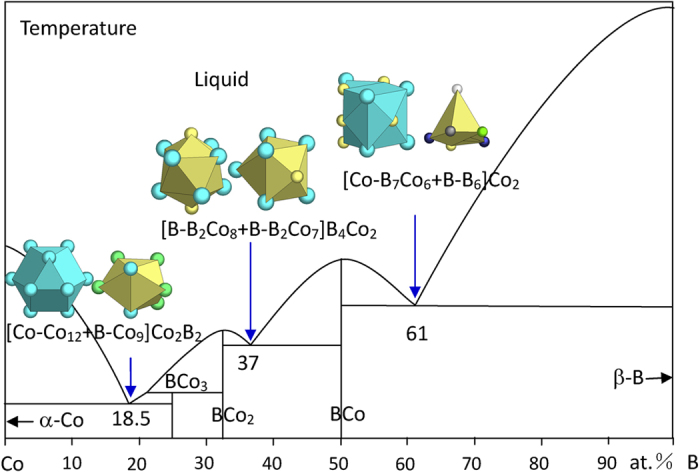
Interpretation of B-Co eutectic points. The dual-cluster formulas come from eutectic phases α-Co-BCo_3_, BCo_2_-BCo, and BCo-β-B.

The B-Co system contains three eutectic points, exemplifying three major types, metal-compound, compound-compound, and compound-boron (Fig. 2). In the present paper, the phase diagrams are all readapted from ref. 24.

The eutectic B_18.5_Co_81.5_ involves eutectic phases α-Co and BCo_3_. α-Co has the Mg structure, which presents a unique twinned cuboctahedron CN12 cluster, typical for the hexagonal close-packed metals. This CN12 [Co-Co_12_] cluster together with CN9 [B-Co_9_] from BCo_3_, plus four B glue atoms, explain the experimental eutectic as


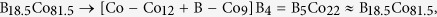
 where the clusters come from Co (Mg) and BCo_3_ (CFe_3_), as shown in Fig. 2 (the cluster configurations are also marked) and Table 1.

The eutectic B_37_Co_63_ involves eutectic phases BCo_2_ and BCo. BCo_2_ is of the AlCu_2_ type and presents two clusters, B-centered octahedral antiprism CN10 [B-B_2_Co_8_] 
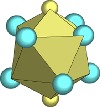
 and Co-centered CN15 [Co-B_3_Co_12_] 
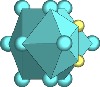
. The phase formulas expressed by the two clusters are [B-Co_2_] and [Co-B_0.5_], respectively. The principal cluster is then the former cluster [B-B_2_Co_8_] that produces a larger phase formula. This is also a common cluster type in eutectic formulas.

BCo is of the BFe structure and presents two types of clusters, capped trigonal prism CN9 [B-B_2_Co_7_] 
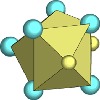
 and CN13 [Co-B_7_Co_6_] 
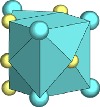
. The [B-B_2_Co_7_] cluster has the same geometry as [B-Co_9_], but with two of the three capping Co atoms replaced by B atoms. The phase formulas expressed by the two clusters are respectively [B-Co] and [Co-B], both containing two atoms. Both are taken as the principal clusters. The eutectic B_37_Co_63_ is explained using both clusters, as shown in Fig. 2 and Table 1:


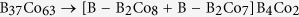
 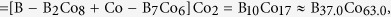 where the clusters come from BCo_2_ (Al_2_Cu) and BCo (BFe).

The eutectic B_61_Co_39_ involves BCo and β-B. Although the boron structures are generally analyzed as based on B_12_ icosahedron, the unique local structure in terms of the nearest-neighbor coordination polyhedron is, however, the pentagonal pyramid [B-B_6_] 
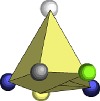
, and the commonly-used B_12_ icosahedron is actually formed by twelve such pyramids enclosing an empty center.

The B-richest eutectic is explained as


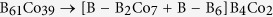
 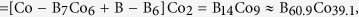 where the clusters come from BCo (BFe) and β-B, as shown in Fig. 2 and Table 1. Again the two principal clusters from BCo are both used.

The deviations between the calculated and experimental compositions are respectively (100*5/22–18.5)*√2 ≈ 0.0 (a scale of √2 should be used to reach the real composition distance), (100*10/27–37.0)*√2 ≈ 0, (100*14/23–61)*√2 ≈ −0.1 at.% B (the negative sign means that the calculated composition is slightly B leaner than the experimental one), which are well below the normal experimental accuracy of about 1 at. %. The deviations are listed in Table 1 and are not mentioned below because they all lie within 1 at.% accuracy.

In reaching the final eutectic formulas using the two principal clusters from relevant eutectic phases, different combinations of glue atoms have been attempted and in general there is only one solution that fits the experimental eutectic point. For instance, the principal clusters [Co-Co_12_] from eutectic phase Co and [B-Co_9_] from eutectic phase BCo_3_, in combination with different glue atoms (B,Co)_2,4,6_, produce dual cluster formulas [Co-Co_12_ + B-Co_9_](B,Co)_2,4,6_, with their total number of atoms per unit formula being 25, 27, and 29. These numbers times the experimental eutectic composition result in 25*(B_18.5_Co_81.5_) = B_4.625_Co_20.375_, 27*(B_18.5_Co_81.5_) = B_4.995_Co_22.005_, and 29*(B_18.5_Co_81.5_) = B_5.365_Co_23.635_. The second formula, being the closest to an integer form B_5_Co_22_, is adopted to explain the eutectic, B_5_Co_22_ ≈ B_18.5_Co_81.5_. The other two are too much deviated from any integer forms by about 2 at.% B.

In the next section, well-established boron-containing binary eutectics are analyzed in the same manner, and they are all well explained by dual-cluster eutectic formulas using the principal clusters derived from relevant eutectic phases.

## Dual cluster formulas of boron-containing binary eutectics

The boron-containing systems are analyzed in the sequence of element groups in the periodic table.

1) IIA element Be

Among the B-IIA binary systems, only B-Be presents a eutectic-type phase diagram at B_11.4_Be_88.6_, bounded by eutectic phases α-Be and BBe_4_. α-Be has the same Mg structure as α-Co, and its cluster type is the CN12 twinned cuboctahedron [Be-Be_12_]. BBe_4_ presents four clusters, [B-Be_9_], [Be-B_4_Be_6_], [Be-BBe_12_], and [Be-B_2_Be_10_]. The corresponding phase formulas are respectively [B-Be_4_], [Be-BBe_2_]Be, [Be-BBe_2_]Be, and [Be-B_0.5_B_1_]. The first cluster [B-Be_9_] is chosen as the principal cluster because it generates the largest phase formula of five atoms, [B-Be_4_], without glue atoms. The eutectic point is explained with

**Figure 3:**
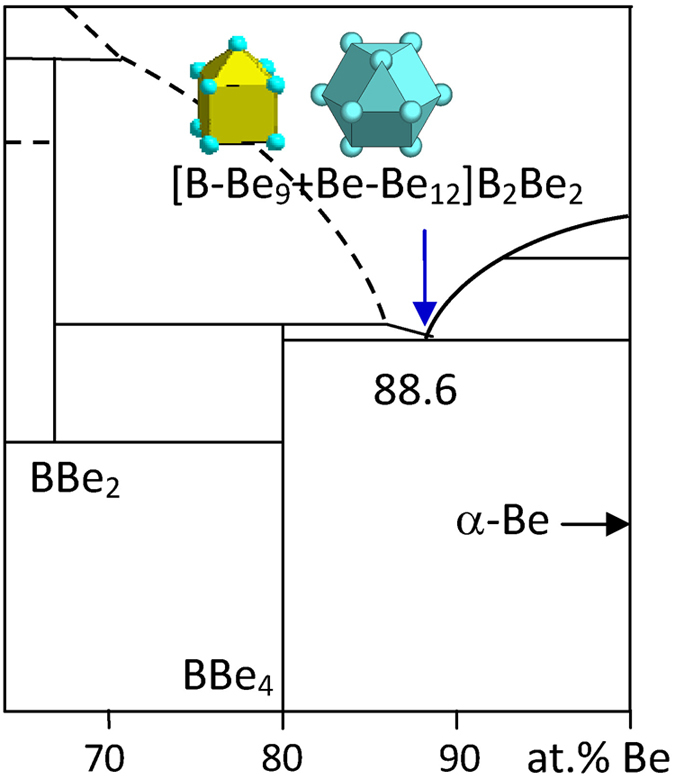
Interpretation of the B-Be eutectic point. The dual cluster formula comes from eutectic phases BBe_4_ and α-Be.

B_11.4_Be_88.6_ → [B-Be_9_ + Be-Be_12_]B_2_Be_2_ = B_3_Be_24_ ≈ B_11.1_Be_88.9_, where the clusters come from BBe_4_ and α-Be (Mg), as shown in Fig. 3 and Table 1.

2) IIIA elements Sc and Y

α-(Sc,Y) of the Mg structure presents the CN12 twinned octahedral cluster.

**Figure 4:**
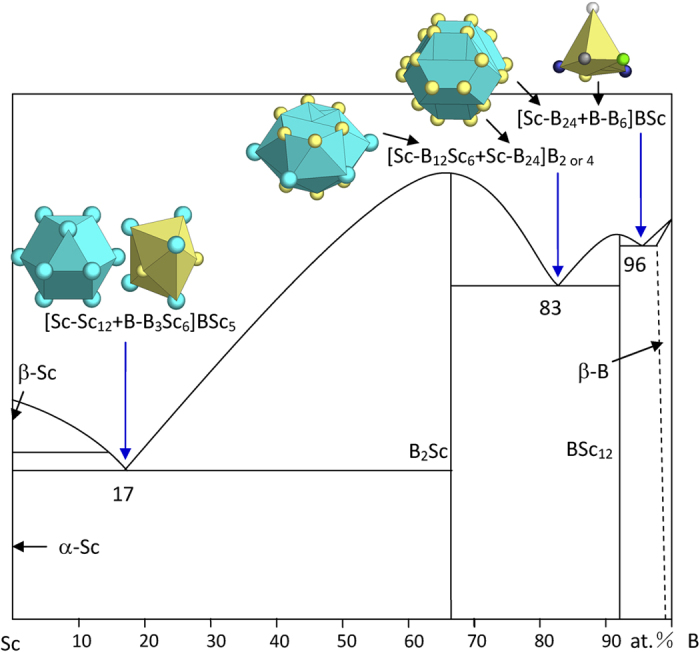
Interpretation of B-Sc eutectic points. The dual cluster formulas come from eutectic phases α-Sc-B_2_Sc, B_2_Sc-BSc_12_, and BSc_12_-β-B. Notice that two clusters from B_2_Sc are both used: [B-B_3_Sc_6_] for the Sc-richer eutectic B_17_Sc_83_ and [Sc-B_12_Sc_6_] for the B-richer eutectic B_83_Sc_17_.

B_2_Sc is of the AlB_2_ structure and presents two clusters, [Sc-B_12_Sc_6_] and capped trigonal prism [B-B_3_Sc_6_]. The phase formulas expressed by the two clusters are [Sc-B_2_] and [B-Sc_0.5_]. In transforming from the initial cluster [Sc-B_12_Sc_6_] of 21 atoms to the final phase formula [Sc-B_2_] of 3, the size reduction rate is 3/21 = 0.14, which is comparable to that from [B-B_3_Sc_6_] to [B-Sc_0.5_] with a size reduction rate of 1.5/10 = 0.15, signifying that both can be the principal clusters. The eutectic B_17_Sc_83_ is explained by [B-B_3_Sc_6_] from BSc_2_ and [Sc-Sc_12_] from Sc (Mg), as shown in Fig. 4 and Table 1:


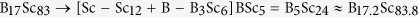


The B-rich cluster [Sc-B_12_Sc_6_], containing too much B, cannot explain this eutectic point.

The B_12_Sc phase is of the B_12_U structure and presents two clusters, [B-B_5_] (square pyramid) and [Sc-B_24_]. The phase formulas are respectively [B]Sc_1/12_ and [Sc-B_12_], so that the latter cluster is selected as the principal cluster for the largest phase formula without glue atoms.

The B-rich eutectic B_83_Sc_17_ is explained with this [Sc-B_24_], together with [Sc-B_12_Sc_6_] (B-rich) from B_2_Sc (Fig. 4 and Table 1), expressed by

B_83_Sc_17_ → [Sc-B_12_Sc_6_ + Sc-B_24_]B_2_ = B_38_Sc_8_ ≈ B_82.6_Sc_17.4_ or by [Sc-B_12_Sc_6_ + Sc-B_24_]B_4_ = B_40_Sc_8_ ≈ B_83.3_Sc_16.7_.

The B-richest eutectic B_94_Sc_6_ is explained with:

B_94_Sc_6_ →[Sc-B_24_ + B-B_6_]BSc = B_32_Sc_2_ ≈ B_94.1_Sc_5.9_, where the clusters come from B_12_Sc (B_12_U) and β-B (Fig. 4 and Table 1).

New structures encountered in the B-Y system are B_4_Y and B_66_Y.

The determination of principal cluster [Y-B_18_Y_5_] in B_4_Y (B_4_Th structure) has already been discussed earlier.

**Figure 5:**
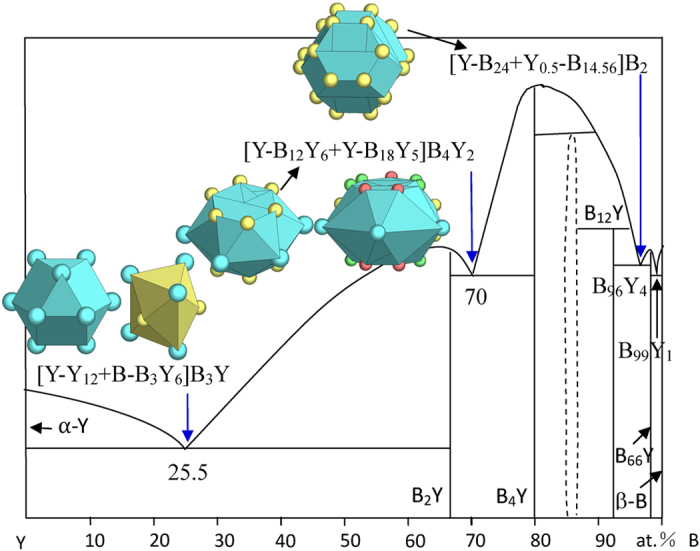
Interpretation of B-Y eutectic points. The dual-cluster formulas come from eutectic phases α-Y-B_2_Y, B_2_Y-B_4_Y, and B_12_Y-B_66_Y.

The B_66_Y phase contains 1936 atoms in its unit cell, and the cluster centered by Y is taken as the principal cluster because it generates a phase formula of the largest size. Geometrically this cluster is expressed as [Y-YB_24_]. However, due to the complicated site occupancies, the real coordination environment is [Y_0.5_- B^13^_4*0.28_Y_0.5_B^6^_2*1_B^5^_2*1_B^12^_4*0.65_B^10^_4*1_B^11^_4*0.71_] = [Y_0.5_-B_14.56_], where the superscripts indicate the atomic sites as in Pearson’s handbook. This cluster configuration is not shown for its complexity in Fig. 5, where all the B-Y eutectics are explained (also in Table 1).

Like in B-Sc, the Y-richest eutectic B_25.5_Y_74.5_ is explained with [Y-Y_12_] from α-Y (Mg) and the Y-rich [B-B_3_Y_6_] cluster from B_2_Y (AlB_2_):


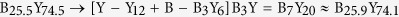


Though the clusters are of the same types as the B-Sc case, the glue atoms are different. Actually, as will be further shown, eutectics are rarely the same, even for those of the nearly same chemistries and cluster structures.

The eutectic B_70_Y_30_ is explained with:

B_70_Y_30_ → [Y-B_12_Y_6_ + Y-B_18_Y_5_]B_4_Y_2_ = B_34_Y_15_ ≈ B_69.4_Y_30.6_, where the two clusters are from the eutectic phases B_2_Y (the B-rich cluster is used) and B_4_Y.

The B_96_Y_4_ eutectic is explained with [Y-B_24_] from B_12_Y and [Y_0.5_-B_14.56_] from B_99_Y:


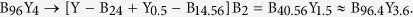
The B-richest eutectic B_99_Y_1_, being located very close to the B end, is not explained.

Again it is noticed that eutectic formulas are rarely identical, despite very similar outer-electron configurations and cluster types in both systems. It is also worth pointing out that the eutectic formation generally involves certain cluster matching rules for the two types of clusters of different geometries and chemistries, which constitutes another general property of eutectic formulas.

3) IVA elements Zr and Hf

These systems are characterized by the presence of two terminal eutectics close to the two elemental ends. The B-Ti and all the B-rich eutectics, too close to phase diagram terminals, cannot be explained by any dual cluster formulas. The B_14_Zr_86_ and B_13_Hf_87_ eutectic points will be dealt with here. The former one is bounded by BCC β-Zr (W) and B_2_Zr (AlB_2_). The β-Zr structure presents a unique CN14 rhombi dodecahedral cluster [W-W_14_], typical for BCC and its superstructures. This cluster, together with the Zr-rich [B-B_3_Zr_6_] cluster from B_2_Zr (AlB_2_), explain the experimental eutectic

**Figure 6:**
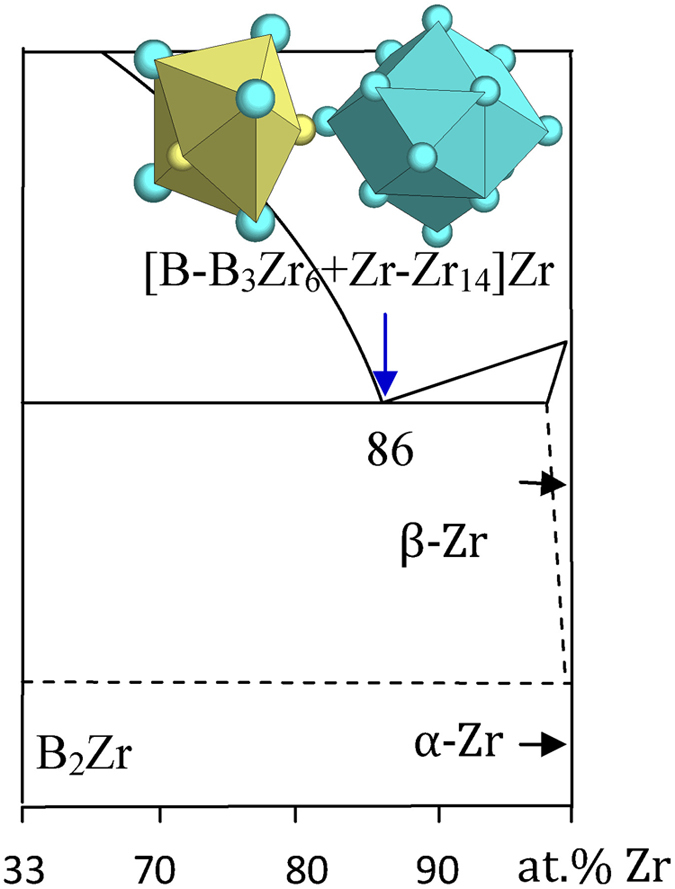
Interpretation of the B-Zr eutectic point. The dual cluster formula comes from eutectic phases B_2_Zr and β-Zr.

B_14_Zr_86_ → [B-B_3_Zr_6_ + Zr-Zr_14_]Zr_4_ = B_4_Zr_25_ ≈ B_13.8_Zr_86.2_ (Fig. 6 and Table 1).

**Figure 7:**
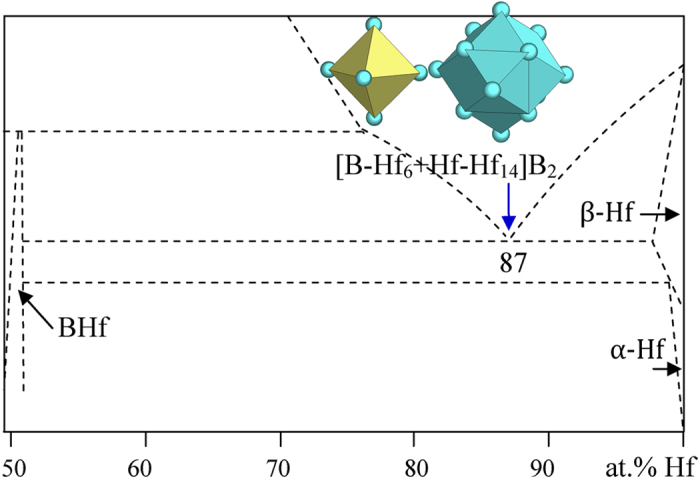
Interpretation of the B-Hf eutectic point. The dual cluster formula comes from eutectic phases BHf and β-Ti.

The B_13_Hf_87_ eutectic is bounded by β-Hf (W) and BHf (ClNa), the latter being characterized by two octahedral clusters [B-Hf_6_] and [Hf-B_6_]. Both clusters, giving phase formulas of the same size of two atoms, but the former one is more densely packed (B being a small atom in the octahedral interstice site of Hf) and is taken as the principal cluster:

B_13_Hf_87_ → [B-Hf_6_ + Hf-Hf_14_]B_2_ = B_3_Hf_21_ = B_12.5_Hf_87.5_ (Fig. 7 and Table 1).

Notice that, again, despite of the extreme similarities between Zr and Hf, even when their eutectics are quite near each other, they corresponds to different eutectic phases and henceforth different dual cluster formulas.

4) VA elements V, Nb, and Ta

These VA elements, like the IV ones, generally form terminal eutectics, except Ta that also forms an intermediate eutectic in the middle part of the phase diagram. New structure types involved are B_2_(V,Nb)_3_, B(V,Nb), and B_4_Nb_3_.

B_2_(V,Nb)_3_ is of the Si_2_U_3_ structural type and presents rhombi dodecahedron [(V,Nb)-B_4_(V,Nb)_10_], capped trigonal prism [B-B(V,Nb)_8_], and [(V,Nb)-B_6_(V,Nb)_11_]. The phase formulas expressed by these clusters are respectively [(V,Nb)-B_2_(V,Nb)_2_], [B-(V,Nb)_1.5_], and [(V,Nb)-B_0.5_(V,Nb)]. The first cluster [B-B(V,Nb)_8_] should be the principal one for its largest phase formula size of five atoms.

The B_4_(Nb,Ta)_3_ phases are of the B_4_Ta_3_ structure and presents four clusters, two capped trigonal prisms [B-B_2_(Nb,Ta)_7_] and [B-B_3_(Nb,Ta)_6_], [(Nb,Ta)-B_7_(Nb,Ta)_8_], and [(Nb,Ta)-B_12_(Nb,Ta)_6_]. The phase formulas are respectively two [B-B(Nb,Ta)_1.5_], [(Nb,Ta)-B_2_(Nb,Ta)_0.5_], and [(Nb,Ta)-B_4_(Nb,Ta)_2_], absent of glue atoms. The last cluster [(Nb,Ta)-B_12_(Nb,Ta)_6_] is chosen as the principal cluster for the largest phase formula generated. This is also the only B-rich cluster and is the closest to the phase composition.

B(Nb,Ta) is of the BCr structure and presents [B-B_2_(Nb,Ta)_7_] and [(Nb,Ta)-B_7_(Nb,Ta)_10_] clusters. The relevant phase formulas are [B-(Nb,Ta)] and [(Nb,Ta)-B], respectively. Like in the BFe structure type, both can be used in interpreting eutectics.

**Figure 8:**
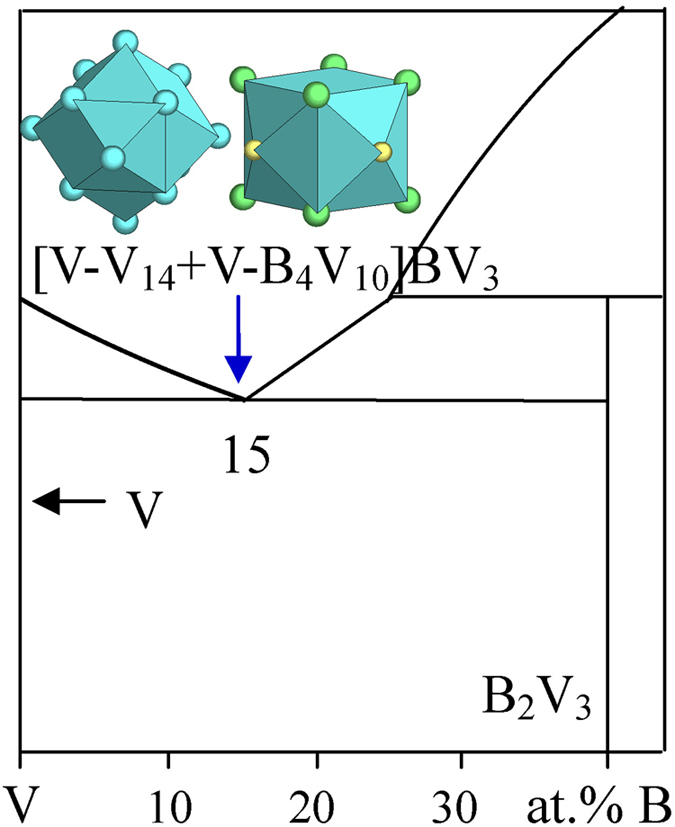
Interpretation of the B-V eutectic point. The dual cluster formula comes from eutectic phases V and B_2_V_3_.

**Figure 9:**
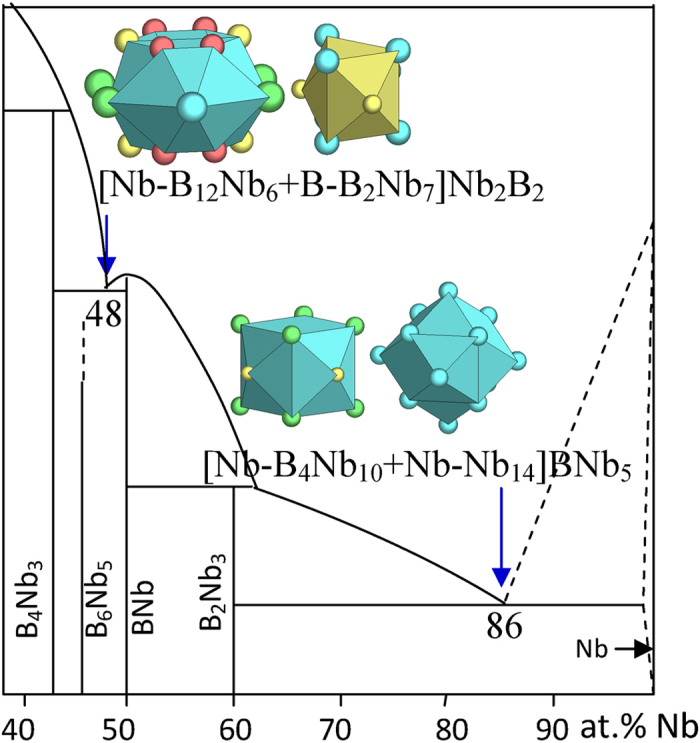
Interpretation of B-Nb eutectic points. The dual cluster formulas come from eutectic phases Nb-B_2_Nb and B_4_Nb_3_-BNb.

**Figure 10:**
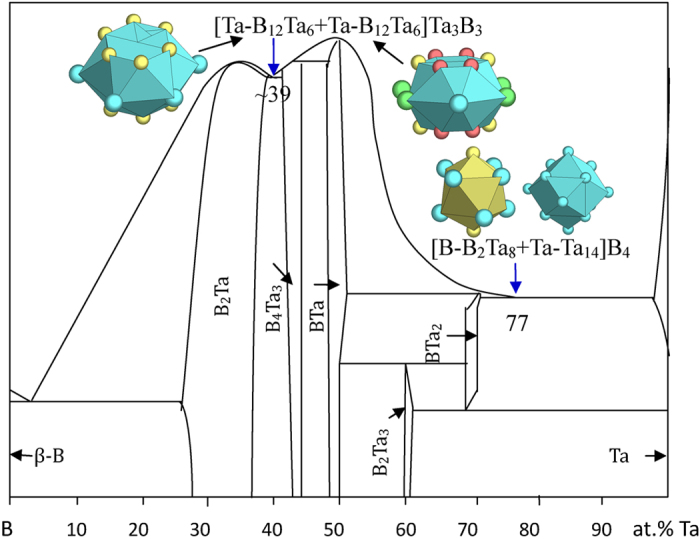
Interpretation of B-Ta eutectic points. The dual cluster formulas come from eutectic phases BTa_2_-Ta and B_2_Ta-B_4_Ta_3_.

The B-(V,Nb,Ta) eutectics are well explained using the principal clusters from respective eutectic phases (Figs 8, 9, 10 and Table 1):

B_15_V_85_ → [V-V_14_ + V-B_4_V_10_]BV_3_ = B_5_V_29_ ≈ B_14.7_V_85.3_ (Fig. 8), where the clusters come from V (W) and B_2_V_3_ (Si_2_U_3_);

B_14_Nb_86_ → [Nb-B_4_Nb_10_ + Nb-Nb_14_]BNb_5_ = B_5_Nb_31_ ≈ B_13.9_Nb_86.1_ (Fig. 9), where the clusters come from B_2_Nb_3_ (Si_2_U_3_) and Nb (W);

B_52_Nb_48_ → [Nb-B_12_Nb_6_ + B-B_2_Nb_7_]Nb_2_B_2_ = B_17_Nb_16_ ≈ B_51.5_Nb_48.5_ (Fig. 9), where the clusters come from B_4_Nb_3_ (B_4_Ta_3_) and BNb (BCr);

B_23_Ta_77_ → [B-B_2_Ta_8_ + Ta-Ta_14_]B_4_ = B_7_Ta_23_ ≈ B_23.3_Ta_76.7_ (Fig. 10), where the clusters come from BTa_2_ (Al_2_Cu) and Ta (W);

B_61_Ta_39_ → [Ta-B_12_Ta_6_ + Ta-B_12_Ta_6_]Ta_3_B_3_ = B_27_Ta_17_ ≈ B_61.4_Ta_38.6_ (Fig. 10), where the clusters come from B_2_Ta (AlB_2_) and B_4_Ta_3_.

The cluster [B-B_3_Ta_6_] from B_2_Ta (AlB_2_) cannot give any solutions to the last eutectic. This is also the rare example where the dual cluster formula is constructed from two identical clusters, which indicates that the glue atom matching can also be important. It is noted that the glue atoms Ta_3_B_3_ cannot be evenly assigned to the identical cluster [Ta-B_12_Ta_6_], so that the resultant cluster formulas for the two liquid subunits are never identical, which is also a common feature for eutectics.

5) VIA elements Cr, Mo, and W

**Figure 11:**
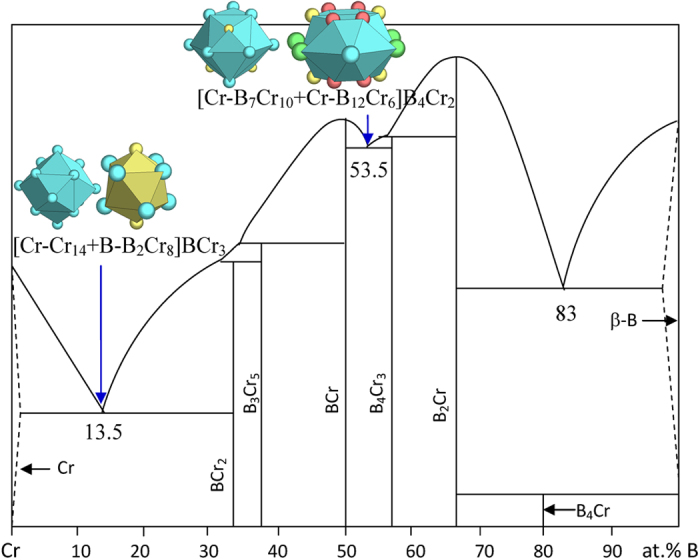
Interpretation of B-Cr eutectic points. The dual cluster formulas come from eutectic phases Cr-BCr_2_ and BCr-B_4_Cr_3_.

No new structure type appears in the B-Cr system. The eutectics are all well explained (Fig. 11 and Table 1):

B_13.5_Cr_86.5_ → [Cr-Cr_14_ + B-B_2_Cr_8_]BCr_3_ = B_4_Cr_26_ ≈ B_13.3_Cr_86.7_, where the clusters come from Cr (W) and BCr_2_ (Al_2_Cu);

B_53.5_Cr_46.5_ → [Cr-B_7_Cr_10_ + Cr-B_12_Cr_6_]B_4_Cr_2_ = B_23_Cr_20_ ≈ B_53.5_Cr_46.5_, where the clusters come from BCr and B_4_Cr_3_ (B_4_Ta_3_).

The B-richest eutectic B_83_Cr_17_ cannot be explained with any dual cluster formulas from phase B_2_Cr (AlB_2_) and β-B.

The B-Mo eutectic is explained by

**Figure 12:**
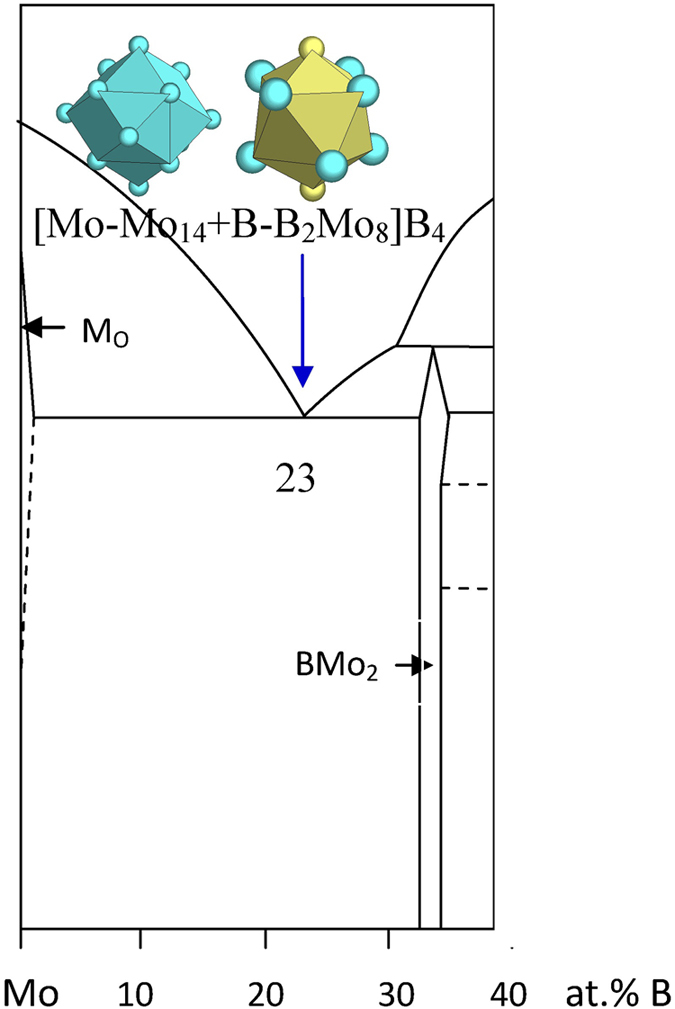
Interpretation of the B-Mo eutectic point. The dual cluster formula comes from eutectic phases Mo and BMo_2_.

B_23_Mo_77_ → [Mo-Mo_14_ + B-B_2_Mo_8_]B_4_ = B_7_Mo_23_ ≈ B_23.3_Mo_76.7_ (Fig. 12 and Table 1), where the clusters come from Mo (W) and BMo_2_ (Al_2_Cu).

B_5_W_2_ has the structure type B_5_Mo_2_, presenting four clusters, [B-B_6_], [B-B_3_], [B-B_6_W_4_], and [W-B_13_]. The relevant phase formulas are respectively [B-B]W_2_B_3_, [B]WB_1.5_, [B-B_0.5_W]B, and [W-B_2.5_]. [W-B_13_] should be the principal clusters for the absence of glue atoms in the largest phase formula.

**Figure 13:**
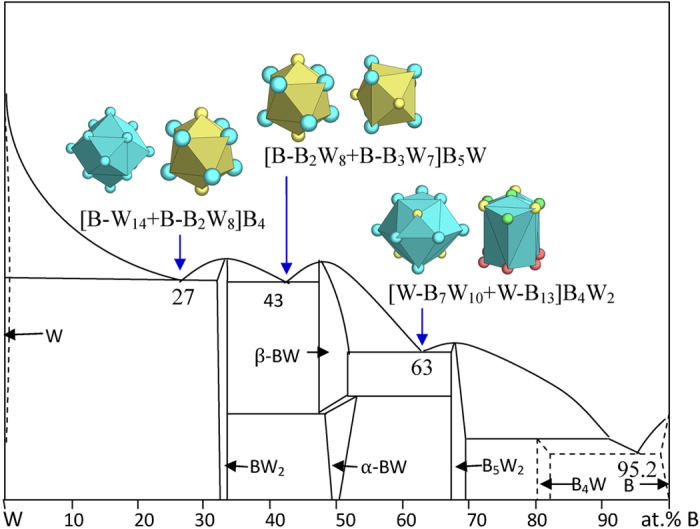
Interpretation of B-W eutectic points. The dual cluster formulas come from eutectic phases Cr - BW_2_, BW_2_ - BW, and BW - B_5_W_2_.

Except the B-richest one, all the B-W eutectics are explained as shown in Fig. 13 and Table 1:

B_27_W_73_ → [B-W_14_ + B-B_2_W_8_]B_4_ = B_8_W_22_ ≈ B_26.7_W_73.7_, where the clusters come from W (W, a B-centered prototype has to be adopted) and BW_2_ (Al_2_Cu);

B_43_W_57_ →[B-B_2_W_8_ + B-B_3_W_7_]B_5_W = B_12_W_16_ ≈ B_42.9_W_57.1_, [B-B_2_W_8_ + W-B_7_W_10_]B_5_W = B_15_W_20_ ≈ B_42.9_W_57.1_, where the clusters come BW_2_ (Al_2_Cu) and β-BW (BCr, the W-rich cluster);

B_63_W_37_ → [B-B_3_W_7_ + W-B_13_]W_2_ = B_17_W_10_ ≈ B_63.0_W_37.0_, [W-B_7_W_10_ + W-B_13_]B_4_W_2_ = B_24_W_14_ ≈ B_63.2_W_36.8_, where the clusters come from β-BW (BCr) and B_5_W_2_ (B_5_Mo_2_).

Notice that in explaining eutectic B_27_W_73_, the cluster is made B-centered on the basis of the pure metal cluster [W-W_14_] in order to give a satisfactory explanation. This is different from all other B-metals systems, where the clusters retain the pure metal forms. At present, we are unable to give a sound explanation for this special case.

6) VIIA element Mn

**Figure 14:**
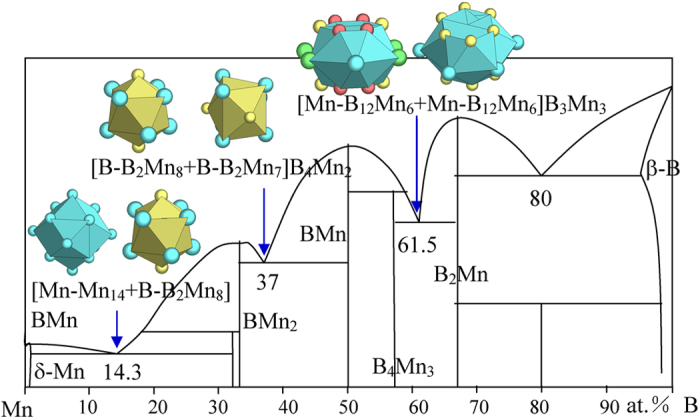
Interpretation of B-Mn eutectic points. The dual cluster formulas come from eutectic phases δ-Mn and BMn_2_, BMn_2_-BMn, and B_4_Mn_3_-B_2_Mn.

No new structure types are encountered in this system. The eutectics are explained as (Fig. 14 and Table 1):

B_14.3_Mn_85.7_ → [Mn-Mn_14_ + B-B_2_Mn_8_]BMn = B_4_Mn_24_ ≈ B_14.3_Mn_85.7_, where the clusters come from δ-Mn (W) and BMn_2_ (Al_2_Cu);

B_37_Mn_63_ → [B-B_2_Mn_8_ + B-B_2_Mn_7_]B_4_Mn_2_ = [B-B_2_Mn_8_ + Mn-B_7_Mn_6_]Mn_2_ = B_10_Mn_17_ ≈ B_37.0_Mn_63.0_, where the clusters come from BMn_2_ (Al_2_Cu) and BMn (BFe);

B_61.5_Mn_38.5_ → [Mn-B_12_Mn_6_ + Mn-B_12_Mn_6_]B_3_Mn_3_ = B_27_Mn_17_ ≈ B_61.4_Mn_38.6_, where the clusters come from B_4_Mn_3_ (B_4_Ta_3_) and B_2_Mn (AlB_2_).

The last one presents another example where two identical clusters are involved, similar to B_61_Ta_39_. The B-richest B_80_Mn_20_ is not explained.

7) VIIIA elements Fe, Co, Ni, and Pd

New structure types are γ-Fe, monoclinic B_3_Ni_4_, orthorhombic B_3_Ni_4_, and B_2_Pd_5_.

γ-Fe (FCC, Cu type) is characterized by a unique cuboctahedral cluster [Fe-Fe_12_], typical for FCC metals.

**Figure 15:**
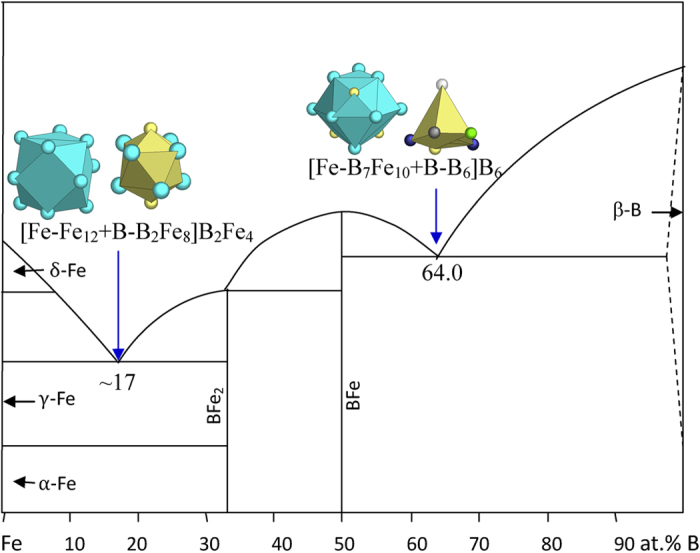
Interpretation of B-Fe eutectic points. The dual cluster formulas come from eutectic phases Fe-BFe_2_ and BFe-B.

The B-Fe eutectics are explained below (Fig. 15 and Table 1):

B_17_Fe_83_ → [Fe-Fe_12_ + B-B_2_Fe_8_]B_2_Fe_4_ = B_5_Fe_25_ ≈ B_16.7_Fe_83.3_, where the clusters come from γ-Fe (Cu) and BFe_2_ (AlCu_2_);

B_64.0_Fe_36.0_ → [Fe-B_7_Fe_10_ + B-B_6_]B_6_ = B_20_Fe_11_ ≈ B_64.5_Fe_35.5_, where the clusters come from BFe (BFe) and β-B.

The m-B_3_Ni_4_ phase contains four clusters, octahedral antiprism [B-B_2_Ni_8_], capped trigonal prism [B-B_2_Ni_7_], [Ni-B_6_Ni_10_], and [B-B_5_Ni_10_]. The phase formulas out of these clusters are [B-B_2_Ni_4_], [B-B_0.5_Ni_2_], [Ni-B_1.5_Ni], and [Ni-B_1.5_Ni]. The [B-B_2_Ni_8_] cluster that leads to the largest formula [B-B_2_Ni_4_] of seven atoms is taken as the principal cluster.

The orthorhombic B_3_Ni_4_ phase contains seven clusters, [B-Ni_9_], [B-B_2_Ni_7_], [B-B_2_Ni_7_], [Ni-B_4_Ni_10_], [Ni-B_6_Ni_11_], [Ni-B_7_Ni_10_], and [Ni-B_6_Ni_11_], which give respective phase formulas [B-Ni_4_]B_2_, [B-Ni_3_]B_2_Ni, [B-Ni_3_]B_2_Ni, [B-BNi_4_]B, [Ni-B_3_Ni_3_], [Ni-B_3_Ni_3_], and [Ni-B_2_Ni_3_]B. The clusters [Ni-B_6_Ni_11_] and [Ni-B_7_Ni_10_] are selected as the principal clusters for the absence of glue atoms in their respective phase formulas.

**Figure 16:**
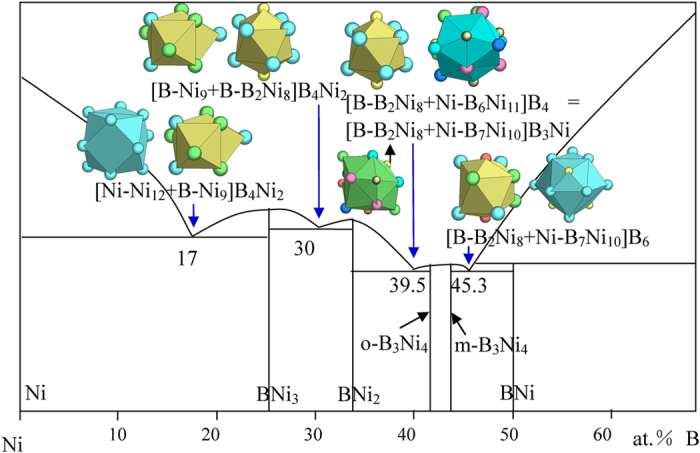
Interpretation of B-Ni eutectic points. The dual cluster formulas come from eutectic phases Ni-BNi_3_, BNi_3_-BNi_2_, BNi_2_-o-B_3_Ni_4_, and m-B_3_Ni_4_-BNi.

The B-Ni eutectics are explained as (Fig. 16 and Table 1):

B_17_Ni_83_ →[Ni-Ni_12_ + B-Ni_9_]B_4_Ni_2_ = B_5_Ni_24_ ≈ B_17.2_Ni_82.8_, where the clusters come from Ni (Cu) and BNi_3_ (CFe_3_);

B_30_Ni_70_ →[B-Ni_9_ + B-B_2_Ni_8_]B_4_Ni_2_ = B_8_Ni_19_ ≈ B_29.6_Ni_70.4_, where the clusters come from BNi_3_ (CFe_3_) and BNi_2_ (AlCu_2_);

B_39.5_Ni_60.5_ →[B-B_2_Ni_8_ + Ni-B_6_Ni_11_]B_4_ = [B-B_2_Ni_8_ + Ni-B_7_Ni_10_]B_3_Ni = B_13_Ni_20_ ≈ B_39.4_Ni_60.6_, where the clusters come from BNi_2_ (AlCu_2_) and o-B_3_Ni_4_ (both principal clusters are used);

B_45.3_Ni_54.7_ → [B-B_2_Ni_8_ + Ni-B_7_Ni_10_]B_6_ = B_16_Ni_19_ ≈ B_45.7_Ni_54.3_, where the clusters come from m-B_3_Ni_4_ and BNi (BCr).

**Figure 17:**
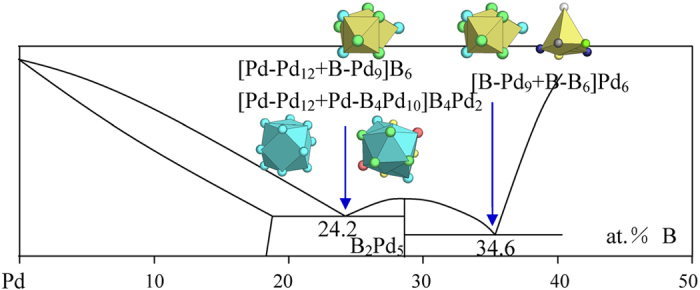
Interpretation of B-Pd eutectic points. The dual cluster formulas come from eutectic phases Pd-B_5_Pd_2_ (or BPd_3_) and BPd_3_-B.

The B_2_Pd_5_ structure presents four clusters, capped trigonal prism [B-Pd_9_], [Pd-B_4_Pd_12_], [Pd-B_4_Pd_10_], and [Pd-B_3_Pd_11_]. The phase formulas expressed by these clusters are respectively [B-Pd_2.5_], [Pd-BPd_1.5_], [Pd-B_2_Pd_4_], and [Pd-BPd_1.5_]. Among them, [Pd-B_4_Pd_10_] generates the largest phase formula and has a cluster reduction rate of 7/15 ≈ 0.47. It is noticed that [B-Pd_9_] generates a smaller phase formula of 3.5 atoms, but its cluster reduction rate of 3.5/9 ≈ 0.39 is only slightly below that of [Pd-B_4_Pd_10_]. This means that both [Pd-B_4_Pd_10_] and [B-Pd_9_] can be the principal clusters. The latter one should be more favored to explain the Pd-rich eutectic. Actually, the [B-Pd_9_] cluster gives better explanations for the eutectics than the [Pd-B_4_Pd_10_] does, as shown below (Fig. 17 and Table 1):

B_24.2_Pd_75.8_ → [Pd-Pd_12_ + Pd-B_4_Pd_10_]B_4_Pd_2_ = B_8_Pd_26_ ≈ B_23.5_Pd_76.5_, where the clusters come from Pd (Cu) and B_2_Pd_5_ (the composition deviation of −0.9 at.% B being the largest in all the eutectics treated here) or [Pd-Pd_12_ + B-Pd_9_]B_6_ = B_7_Pd_22_ ≈ B_24.1_Pd_75.8_ using the clusters from Pd (Cu) and BPd_3_ (CFe_3_);

B_34.6_Pd_65.4_ → [B-Pd_9_ + B-B_6_]Pd_6_ = B_8_Pd_15_ ≈ B_34.8_Pd_65.2_, where the clusters come from B_3_Pd (CFe_3_) and β-B.

8) IVB element C

Only C forms a eutectic type phase diagram with B. There is a carbon boride solid solution zone with a rough B_4_C empirical composition. The B_13_C_2_ structure presents four clusters, [B-C_2_], tetrahedron [C-B_4_], pentagonal pyramid [B-CB_5_], and pentagonal pyramid [B-B_6_]. It should be noticed that in describing borides, we stick to the principal of nearest-neighbor coordination polyhedron, which leads to the pentagonal pyramids, rather than the B_12_ icosahedron without central atom as normally used in the literature. The phase formula expressed by these clusters are respectively [B-C_2_]B_12_, [C-B_0.5_B_3_]B_3_, [B-B_7/6_C_1/3_], and [B-B]B_1/6_C_1/3_. Among them, the [B-CB_5_] cluster expresses the phase without using glue atoms and is taken as the principal cluster.

Graphite has a layered hexagonal structure, presenting two identical triangular clusters [C-C_3_].

**Figure 18:**
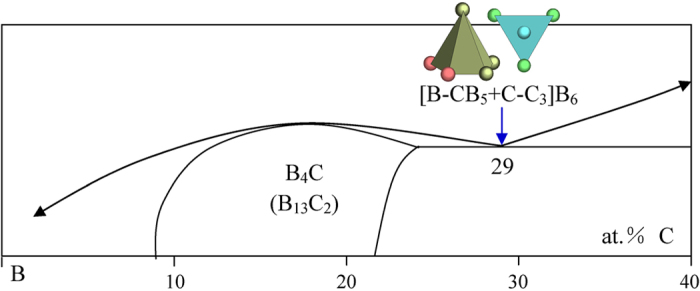
Interpretation of the B-C eutectic point. The dual cluster formula comes from eutectic phases B_13_C_2_ and B.

The only eutectic point is explained with the pentagonal pyramid [B-CB_5_] from B_13_C_2_ and the triangular [C-C_3_] from graphite (Fig. 18):


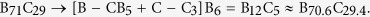


This B-C system exemplifies the eutectic systems possessing covalent bonding, signifying the universality of the present formulism for binary eutectics.

## Dual cluster formulas of some common binary eutectics

Other eutectics are being examined, and the dual cluster formulas are generally preserved but sometimes modifications on clusters are necessary. A few commonly encountered eutectics involving simple structures and pure metals are attempted below.

C_17.3_Fe_82.7_ → [Fe-Fe_12_ + C-Fe_9_]C_4_Fe_2_ = C_5_Fe_24_ ≈ C_17.2_Fe_82.8_, where the clusters are from γ-Fe (Cu) and CFe_3_ (cementite);

Cr_56_Ni_44_ → [Ni-Ni_12_ + Cr-Cr_14_]Cr_4_Ni_2_ = Cr_19_Ni_15_ ≈ Cr_55.9_Ni_44.1_, where the clusters come from Ni (Cu) and Cr (W);

Fe_29.5_Ti_70.5_ → [Ti-Ti_14_ + Fe-Ti_8_Fe_6_]Fe_3_Ti_1_ = [Ti-Ti_14_ + Ti-Fe_8_Ti_6_]Fe_2_Ti_2_ =Fe_10_Ti_24_ ≈ Fe_29.4_Ti_70.6_, where the clusters come from Fe (W) and FeTi (CsCl, CN14 rhombi-dodecahedral cluster).

Au_81.4_Si_18.6_ → [Au-Au_12_ + Si-Au_4_]Si_3_Au_1_ = Au_18_Si_4_ ≈ Au_81.8_Si_18.2_, where the clusters come from Au (Cu) and Si (diamond type structure, assuming Au substitutions on the shell sites of the CN4 tetrahedral cluster);

Sn_85.1_Zn_14.9_ → [Sn-Sn_4_ + Zn-Sn_12_]Zn_2_ = Sn_17_Zn_3_ = Sn_85.0_Zn_15.0_, where the clusters come from β-Sn (grey tin, space group 141) and Zn (Mg, assuming Sn substitutions on the shell sites of the CN12 twinned octahedral cluster);

Al_87.8_Si_12.2_ → [Al-Al_12_ + Si-Al_4_]Al_4_Si_2_ = Al_31_Si_3_ = Al_87.5_Si_12.5_, where the clusters come from Al (Cu) and Si (diamond, assuming Al substitutions at the shell sites of the tetrahedral cluster).

Ag_60.1_Cu_39.9_ → [Ag-Ag_12_ + Ag-Cu_12_]Ag_4_ = Ag_18_Cu_12_ = Ag_60_Cu_40_, where the clusters come from Ag (Cu) and Cu (assuming Ag substitution at the center site).

Al_82.9_Cu_17.1_ → [Al-Al_12_ + Cu-Cu_2_Al_8_]Al_4_Cu_2_ = Al_25_Cu_5_ ≈ Al_83.3_Cu_16.7_, where the clusters come from Al (Cu) and Al_2_Cu.

In all these interpreted eutectic formulas, the dual cluster form is always retained but the compositions of the pure metal cluster are generally adjusted. At present, the general substitution rules in these pure metal clusters are not completely understood. More examples should be analyzed, which will be the objective of our future research.

## Properties of eutectic formulas

Except some terminal eutectics by the B side and all those located extremely close to the other sides, normal eutectics in B-containing binary systems are all well explained with dual cluster formulas formed with clusters from relevant eutectic phases plus appropriate glue atoms of 2, 4, or 6. The eutectic formulas, for B eutectics but not limited to, present the following common properties:

1) Eutectic formulas vary extensively, complicated by different cluster geometries and configurations as well as by the matching of different glue atoms. For instance, two identical pairs of clusters [Co-Co_12_ + B-Co_9_] and [Ni-Ni_12_ + B-Ni_9_], after being matched with different glue atoms, respectively B_4_ and B_4_Ni_2_, point to two different eutectic compositions B_18.5_Co_81.5_ and B_17_Ni_83_. In all the 35 eutectics dealt with here, only two identical eutectic formulas are encountered, B_7_(Ta,Mo)_23_ and B_27_(Ta,Mn)_17_.

2) A eutectic formula is not constructed from two identical subunits. Even for eutectic compositions expressed with identical clusters like B_27_(Ta,Mn)_17_ = [(Ta,Mn)-B_12_(Ta,Mn)_6_ + (Ta,Mn)-B_12_(Ta,Mn)_6_]B_3_Mn_3_, the two subunits cannot be made identical, because the glue atoms B_3_M_3_ cannot be evenly assigned to each cluster.

3) Eutectic formulas are generally formed from two distinct types of clusters.

Many cluster types are involved. Common ones are CN9 capped trigonal prisms, CN10 octahedral antiprisms, CN12 octahedra or twinned octahedra, and CN14 rhombi dodecahedra. This fact indicates that cluster matching plays a dominating and yet unknown role in stabilizing a eutectic liquid, and the distinctly different clusters lead to two eutectic phases of large composition differences. The exceptions are found only for [(Ta,Mn)-B_12_(Ta,Mn)_6_ + (Ta,Mn)-B_12_(Ta,Mn)_6_](Ta,Mn)_3_B_3_, where the two clusters are identical so that the two subunits should be stabilized by different but unknown attributions of glue atoms to each cluster.

Regarding the cluster matching rule, the CN12 clusters from FCC and HCP metals are generally associated with CN9 capped trigonal prisms such as [B-(Be,Co,Ni,Pd)_9_] and [B-B_3_(Sc,Y)_6_]. The CN14 clusters from the BCC W structure are matched to more varieties of clusters, but the occurrence of the CN10 octahedral antiprism clusters is the most frequent.

4) Simple integer ratios

In confirmation of Kokandale’s eutectic puzzle that eutectics occur near simple composition ratios^10^, the present B-containing binary eutectics are especially abundant by composition ratios of 1:6 (the first number representing B number), 1:5, 1:3, 3:5, 3:2, and 5:2 (Table 1), though more eutectics should be treated in order to give a better statistic account of the eutectic distribution.

By unveiling the cluster-based formulism, we have actually developed a new tool for the description of liquid structures, focusing only on their characteristic short-range-order units. Experimental validation of the cluster-based eutectic structures can now be envisaged, which has long been hindered by the lack of a suitable short-range-order structural model. Also, our approach and the formulism thereof may provide a practical composition design method for multi-element eutectic alloys via substitutions of binary eutectic formulas by similar elements.

In conclusion, eutectic structure and composition rule have been addressed using the cluster-plus-glue-atom model for the description of short-range-order structures. It is assumed that a eutectic liquid consist of two different subunits issued from the relevant eutectic phases, each being expressed by the cluster formula for ideal metallic glasses, i.e., [cluster](glue atom)_1 or 3_. Such a dual cluster formulism is well validated in B-containing eutectics (except those located by the extreme phase diagram ends). The dual cluster formulas vary extensively and are always composed of different subunits. They are generally formed with two distinctly different cluster types, with special cluster matching rules such as cuboctahedron with capped trigonal prism and rhombi-dodecahedron with octahedral antiprism.

## Additional Information

**How to cite this article**: Ma, Y. P. *et al.* Composition formulas of binary eutectics. *Sci. Rep.* **5**, 17880; doi: 10.1038/srep17880 (2015).

## Figures and Tables

**Table 1:** Eutectic compositions in boron-containing binary systems and their interpretations using dual cluster formulas from eutectic phases.

**Exp. eutectics**	**Cal. Eutectics (deviation in at.% B)**	**Eutectic phases (structural types)**	**Dual cluster formulas and near-integer ratios**
B_11.4_Be_88.6_	B_11.1_Be_88.9_ (−0.4)	BBe_4_ (BBe_4_) + α-Be (Mg)	[B-Be_9_ + Be-Be_12_]B_2_Be_2_ = B_3_Be_24_= BBe_8_
B_17_Sc_83_	B_17.2_Sc_83.8_ (0.3)	α-Sc (Mg) + B_2_Sc (AlB_2_)	[Sc-Sc_12_ + B-B_3_Sc_6_]BSc_5_ = B_5_Sc_24_= BSc_4.8_
B_83_Sc_17_	B_82.6_Sc_17.4_ (−0.6)	B_2_Sc (AlB_2_) + B_12_Sc (B_12_U)	[Sc-B_12_Sc_6_ + Sc-B_24_]B_2_ = B_38_Sc_8_≈ B_4.7_Sc
	B_83.3_Sc_16.7_ (0.5)		[Sc-B_12_Sc_6_ + Sc-B_24_]B_4_ = B_40_Sc_8_ = B_5_Sc
B_94_Sc_6_	B_94.1_Sc_5.9_ (0.2)	B_12_Sc (B_12_U) + β-B	[Sc-B_24_ + B-B_6_](BSc) = B_32_Sc_2_= B_16_Sc
B_25.5_Y_74.5_	B_25.9_Y_74.1_ (0.6)	α-Y (Mg) + B_2_Y (AlB_2_)	[Y-Y_12_ + B-B_3_Y_6_]B_3_Y = B_7_Y_20_ ≈ BY_2.9_
B_70_Y_30_	B_69.4_Y_30.6_ (−0.9)	B_2_Y (AlB_2_) + B_4_Y (B_4_Th)	[Y-B_12_Y_6_ + Y-B_18_Y_5_]B_4_Y_2_ = B_34_Y_15_≈ B_4_Y_9.1_
B_96_Y_4_	B_96.4_Y_3.6_ (0.6)	B_12_Y (B_12_U) + B_66_Y (B_66_Y)	[Y-B_24_ + Y_0.5_-B_14.56_]B_2_ = B_40.56_Y_1.5_≈ B_27.0_Y
B_14_Zr_86_	B_13.8_Zr_86.2_ (−0.3)	B_2_Zr (AlB_2_) + β-Zr (W)	[B-B_3_Zr_6_ + Zr-Zr_14_]Zr_4_ = B_4_Zr_25_ ≈ BZr_6.2_
B_13_Hf_87_	B_12.5_Zr_87.5_ (−0.7)	BHf (ClNa) + β-Hf (W)	[B-Hf_6_ + Hf-Hf_14_]B_2_ = B_3_Hf_21_= BHf_7_
B_15_V_85_	B_14.7_V_85.3_ (−0.4)	V (W) + B_2_V_3_ (Si_2_U_3_)	[V-V_14_ + V-B_4_V_10_]BV_3_ = B_5_V_29_≈ BV_5.8_
B_14_Nb_86_	B_13.9_Nb_86.1_ (−0.2)	B_2_Nb_3_ (Si_2_U_3_) + Nb (W)	[Nb-B_4_Nb_10_ + Nb-Nb_14_]BNb_5_ = B_5_Nb_31_≈ BNb_6.2_
B_52_Nb_48_	B_51.5_Nb_48.5_ (−0.7)	B_4_Nb_3_ (B_4_Ta_3_) + BNb (BCr)	[Nb-B_12_Nb_6_ + B-B_2_Nb_7_]Nb_2_B_2_ = B_17_Nb_16_≈ B_1.1_Nb
B_23_Ta_77_	B_23.3_Ta_76.7_ (0.5)	BTa_2_ (Al_2_Cu) + Ta (W)	[B-B_2_Ta_8_ + Ta-Ta_14_]B_4_ = B_7_Ta_23_ ≈ BTa_3.3_ ≈ B_3_Ta_10_
B_61_Ta_39_	B_61.4_Ta_38.6_ (0.5)	B_2_Ta (AlB_2_) + B_4_Ta_3_	[Ta-B_12_Ta_6_ + Ta-B_12_Ta_6_]Ta_3_B_3_ = B_27_Ta_17_ ≈ B_3.2_Ta_2_
B_13.5_Cr_86.5_	B_13.3_Cr_86.7_ (−0.2)	Cr (W) + BCr_2_ (Al_2_Cu)	[Cr-Cr_14_ + B-B_2_Cr_8_]BCr_3_ = B_4_Cr_26_= B_2_Cr_13_
B_53.5_Cr_46.5_	B_53.5_Cr_46.5_ (−0.0)	BCr + B_4_Cr_3_ (B_4_Ta_3_)	[Cr-B_7_Cr_10_ + Cr-B_12_Cr_6_]B_4_Cr_2_ = B_23_Cr_20_≈ B_1.1_Cr
B_23_Mo_77_	B_23.3_Mo_76.7_ (0.5)	Mo (W) + BMo_2_ (Al_2_Cu)	[Mo-Mo_14_ + B-B_2_Mo_8_]B_4_ = B_7_Mo_23_≈ BMo_3.3_≈ B_3_Mo_10_
B_27_W_73_	B_26.7_W_73.3_ (−0.5)	W (W) + BW_2_ (Al_2_Cu)	[B-W_14_ + B-B_2_W_8_]B_4_ = B_8_W_22_ ≈ B_3_W_8_
B_43_W_57_	B_42.9_W_57.1_ (−0.2)	BW_2_ (Al_2_Cu) + β-BW (BCr)	[B-B_2_W_8_ + B-B_3_W_7_]B_5_W = B_12_W_16_= B_3_W_4_ [B-B_2_W_8_ + W-B_7_W_10_]B_5_W = B_15_W_20_= B_3_W_4_
B_63_W_37_	B_63.0_W_37.0_(0.0) B_63.2_W_36.8_ (0.2)	β-BW (BCr) + B_5_W_2_ (B_5_Mo_2_)	[B-B_3_W_7_ + W-B_13_]W_2_ = B_17_W_10_≈ B_5.1_W_3_ [W-B_7_W_10_ + W-B_13_]B_4_W_2_ = B_24_W_14_≈ B_5.1_W_3_
B_14.3_Mn_85.7_	B_14.3_Mn_85.7_ (−0.0)	δ-Mn (W) + BMn_2_ (Al_2_Cu)	[Mn-Mn_14_ + B-B_2_Mn_8_]BMn = B_4_Mn_24_= BMn_6_
B_37_Mn_63_	B_37.0_Mn_63.0_ (0.0)	BMn_2_ (Al_2_Cu) + BMn (BFe)	[B-B_2_Mn_8_ + B-B_2_Mn_7_]B_4_Mn_2_ = [B-B_2_Mn_8_ + Mn-B_7_Mn_6_]Mn_2_ = B_10_Mn_17_= B_3_Mn_5.1_
B_61.5_Mn_38.5_	B_61.4_Mn_38.6_ (−0.2)	B_4_Mn_3_ (B_4_Ta_3_) + BMn_2_ (AlB_2_)	[Mn-B_12_Mn_6_ + Mn-B_12_Mn_6_]B_3_Mn_3_ = B_27_Mn_17_≈ B_3.2_Mn_2_
B_17_Fe_83_	B_16.7_Fe_83.3_ (−0.5)	γ-Fe (Cu) + BFe_2_ (Al_2_Cu)	[Fe-Fe_12_ + B-B_2_Fe_8_]B_2_Fe_4_ = B_5_Fe_25_ = BFe_5_
B_64_Fe_36_	B_64.5_Fe_35.5_ (0.7)	BFe (BFe) + β-B	[Fe-B_7_Fe_10_ + B-B_6_]B_6_ = B_20_Fe_11_≈ B_1.8_Fe
B_18.5_Co_81.5_	B_18.5_Co_81.5_ (0.0)	α-Co (Mg) + BCo_3_ (CFe_3_)	[Co-Co_12_ + B-Co_9_]B_4_ = B_5_Co_22_≈ B_2_Co_9_
B_37_Co_63_	B_37.0_Co_63.0_ (0.0)	BCo_2_ (Al_2_Cu) + BCo (BFe)	[B-B_2_Co_8_ + B-B_2_Co_7_]B_4_Co_2_ = [B-B_2_Co_8_ + Co-B_7_Co_6_]Co_2_ = B_10_Co_17_= B_3_Co_5.1_
B_61_Co_39_	B_60.9_Co_39.1_ (−0.1)	BCo (BFe) + β-B	[B-B_2_Co_7_ + B-B_6_]B_4_Co_2_ = [Co-B_7_Co_6_ + B-B_6_]Co_2_ = B_14_Co_9_≈ B_3.1_Co_2_
B_17_Ni_83_	B_17.2_Ni_82.8_ (0.3)	Ni (Cu) + BNi_3_ (CFe_3_)	[Ni-Ni_12_ + B-Ni_9_]B_4_Ni_2_ = B_5_Ni_24_= B_5_Ni_4.8_
B_30_Ni_70_	B_29.6_Ni_70.4_ (−0.5)	BNi_3_ (CFe_3_) + BNi_2_ (Al_2_Cu)	[B-Ni_9_ + B-B_2_Ni_8_]B_4_Ni_2_ = B_8_Ni_19_ ≈ B_3_Ni_7.1_
B_39.5_N_60.5_	B_39.4_Ni_60.6_ (−0.1)	BNi_2_ (Al_2_Cu) + o-B_3_Ni_4_	[B-B_2_Ni_8_ + Ni-B_6_Ni_11_]B_4_ = [B-B_2_Ni_8_ + Ni-B_7_Ni_10_]B_3_Ni = B_13_Ni_20_≈ B_2_Ni_3.1_
B_45.3_Ni_54.7_	B_45.7_Ni_54.3_ (0.6)	m-B_3_Ni_4_ + BNi (BCr)	[B-B_2_Ni_8_ + Ni-B_7_Ni_10_]B_6_ = B_16_Ni_19_≈ BNi_1.2_
B_24.2_Pd_75.8_	B_24.1_Pd_77.8_ (−0.1)	Pd (Cu) + B_3_Pd (CFe_3_)	[Pd-Pd_12_ + B-Pd_9_]B_6_ = B_7_Pd_22_≈ BPd_3.1_
B_34.6_Pd_65.4_	B_34.8_Pd_65.2_ (0.3)	B_3_Pd (CFe_3_) + β-B	[B-Pd_9_ + B-B_6_]Pd_6_= B_8_Pd_15_ ≈ BPd_1.9_
B_71_C_29_	B_70.6_C_29.4_ (−0.6)	B_13_C_2_(B_13_C_2_) + C(graphite)	[B-CB_5_ + C-C_3_]B_6_ = B_12_C_5_ = B_4.8_C_2_

The deviation between the calculated and the measured compositions are also shown.
